# Owner’s Anthropomorphic Perceptions of Cats’ and Dogs’ Abilities Are Related to the Social Role of Pets, Owners’ Relationship Behaviors, and Social Support

**DOI:** 10.3390/ani13233644

**Published:** 2023-11-24

**Authors:** Esther M. C. Bouma, Arie Dijkstra, Stella Arnt Rosa

**Affiliations:** Department of Social Psychology, University of Groningen, 9712 TS Groningen, The Netherlands; arie.dijkstra@rug.nl (A.D.);

**Keywords:** anthropomorphization, cats, dogs, social abilities, cognitive abilities, emotions, relationship behavior, social role, social support

## Abstract

**Simple Summary:**

People can develop meaningful relationships with companion animals, and they behave towards them in specific ways. For example, they spend time together in close proximity, apologize or talk to them as if they are human, and experience social support from their pets. This study aims to explain these types of interactions in a sample of cat owners and dog owners in the Netherlands. The more pet owners rated the mental abilities of their pet to be similar to those of humans (anthropomorphization), the more they displayed making-up behavior towards their pet, and the stronger they experienced social support from their pet. However, to engage in communication behavior (e.g., petting, kissing, talking) it is not necessary to anthropomorphize the pet. Dog owners anthropomorphize more than cat owners; this might be due to a higher symmetry in social behaviors between humans and dogs. Lastly, the social role of the pet (partly) mediates the association between anthropomorphization and owner behaviors and experience of social support. This mediation effect is more pronounced in cat owners compared to dog owners.

**Abstract:**

Background: For sustainable and healthy relationships with pets, attributing some degree of human abilities to the pet (anthropomorphization) might be necessary. We hypothesize that the tendency to anthropomorphize pet animals is related to relationship behaviors (communication and making up) and the experience of social support. Perceiving the pet in a human social role (e.g., family member or friend) might mediate this relationship. Method: Associations were tested in a mixed sample of cat and dog owners by means of multiple linear regression, moderation, and (moderated) mediation analyses. The differences between cat and dog owners were examined with pet type as the moderator in a moderated mediation analysis. Results: Dog owners anthropomorphize their pets more than cat owners. The social role of the pet mediates the association between anthropomorphization and relationship behavior and social support. The mediation effects were stronger for cat owners than for dog owners. Moreover, our newly developed comparative anthropomorphism measure was a better predictor than the commonly used general anthropomorphism measure (IDAQ).

## 1. Introduction

In the last 30 to 50 years a shift has occurred in our attitudes towards pets, which are more often seen as individuals and sentient beings [1,2] and are no longer perceived as “pets” but as members of the family [3,4]. Social belonging and meaningful relationships are essential for the wellbeing of human beings [5], as humans evolved from ancestors who lived in close social groups, comparable to other primates [6]. Nowadays, pets such as cats and dogs, are kept mostly for companionship [7] rather than for utilitarian purposes; attachment to pets has been shown to be relational rather than attributable [8]. Most owners name their pet, talk to them, play and sleep with them, take their photographs, treat their illnesses, and mourn them when they die [9]. Pets can fill relational voids in peoples’ lives or be an addition to human social support networks [10,11,12,13] and perceiving a companion animal as member of an important social group is related to human wellbeing [14]. Previous studies show that pets can occupy similar social niches as social partners [15], friends [16,17], family members [17,18,19,20], or children [14,17]. Pets can fulfil our human innate needs to love and take care of another being [5,7,10,18,19,20].

However, Epley, Waytz, and Cacioppo [21] proposed that people differ in their human need to be socially connected to other beings. This “sociality motivation” seems to influence people’s tendency to think about animals as if they are human [22]. Seeing our companion animals as small human-like social entities might be related to our tendency to attribute cognitive and social abilities to our pets as this was found in owners of dogs [23] and cats [24]. Being a family member (instead of a mouser) was, for example, associated with a stronger attribution of friendliness and intelligence to cats by their owners [24] and when dog owners thought of their dogs as children, they rated them as more empathic [25]. These findings suggest that people form (human-like) perceptions of their pets’ social role [17,26,27] that might influence their behavior towards the pet.

Besides the perceived social role of the pet, anthropomorphic perceptions of their pets’ social and cognitive abilities may influence their behavior towards the pet. Cognitive abilities refer to (conscious) intellectual activities with regard to individual reality, such as thinking, reasoning, or remembering, and are different from social or emotional abilities. Anthropomorphizing pets’ cognitive abilities may manifest in owners as beliefs about the pet being able to count or to think about the future. Social abilities refer to intellectual activities with regard to social reality [28] and are related to insight into social (strategic) behaviors and social intentions of others. Anthropomorphizing pets’ social abilities may manifest as beliefs about the pet being able to understand human language or to take revenge when disappointed in the owner or experience complex social emotions such as jealousy and shame. Thus, the owners’ perceptions of cognitive and social abilities of their pet may also influence the interactions with their pet.

Thus, the perceived cognitive and social abilities of their pet, and the perceived social role of the pet, are expected to have various outcomes in the relationship. The present study focuses on three relationship outcomes: the presence of making up and communication behaviors and the experience of social support. All three outcomes can only be understood to occur when people have formed certain perceptions of their pet. Making-up behavior is performed when important (human) relationships are under pressure. These behaviors are based on feelings of guilt that motivate to “repair” the relationship [29,30]. Making-up behavior involves apologizing to the animal, for example for being a long time away from the animal or hurting the animal by accident. Communication behavior refers to behaviors with the goal to interact with another, such as talking and touching. Lastly, the experience of social support is a basic aspect of human relationships [31] that also has been shown in relationships with animals [32,33]. The attribution of abilities like “perceptive”, “empathetic”, and “considerate” to pets makes it possible for them to be a source of emotional support and friendship for humans [34,35], and that being among pets can mitigate the effects of stress [35]. One central question in the present study is whether the perceived social role of the pet, and the perceived cognitive and social abilities of their pet are related to these three relationship outcomes.

How anthropomorphizing one’s pet and perceptions of the pets’ social role influence the human–pet relationship can be understood from Greene’s [36,37] “action assembly theory” people’s communication behaviors are based on the activation of behavioral scripts in communication, which are assembled to be used in a specific context, in this case, communication with one’s pet. The perception of the social role of the pet may provide a cognitive structure related to the activation of specific behavioral scripts. For example, when one feels lonely, receiving support from a friend may need different communication behavioral scripts than receiving support from one’s father, which again is different from receiving support from one’s pet. Thus, relevant differences in interlocuters must be acknowledged before the appropriate communication script can be activated; the pet social role perception may provide a guideline for this. These scripts take into account the peculiarities of communication with a cat or dog. For example, it may determine the words used, the tone of voice, and one’s nonverbal communication. Several studies showed that people tend to speak differently to pets (and children and older people) than to adults [38]. Similar to human communication, communication with a pet is based on action–outcome contingencies that are stored in one’s procedural memory and are activated by a need. For example, “When I need social support [need], I approach my pet in a gentle way [action], and it will fulfil my need [outcome]” or “When I stepped on my pet’s tale (and I have the need to “repair” my relationship [need]), I give it some delicious pet food [action], and I (or we) will feel better again [outcome]”.

**Figure 1:**
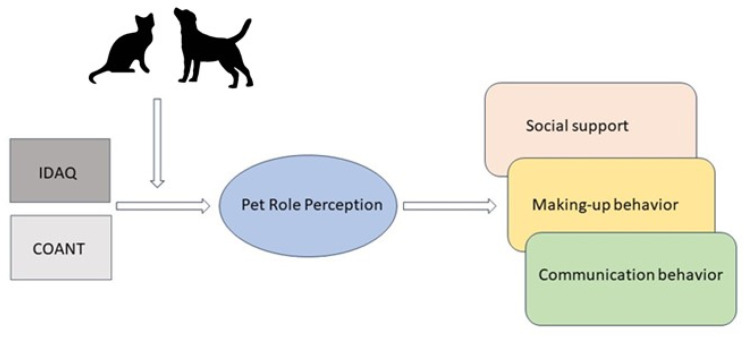
Graphical representation of the moderated mediation analyses. IDAQ = Individual Differences in Anthropomorphism Questionnaire, COANT = comparative anthropomorphisation.

One last assumption in the present study is that the perceptions of the social role of a pet (e.g., a child or a family member) can only develop when people endorse anthropomorphic attributions of abilities; they may perceive and treat the pet in a certain social role only when they assume that the pet has cognitive and social abilities. Taken together, this formulation implies a mediational structure: anthropomorphic attributions of abilities may lead to pet role perceptions, which may influence the three relationship outcomes (making-up behavior, communication behavior, and social support). This theoretical model will be examined in the two companion animals that are the most common in the Western world, cats and dogs [39]. Due to the different nature and social needs of cats and dogs, it is expected that associations will be different in cat owners compared to owners of dogs and will be tested in a moderated mediation analysis (see Figure 1 for graphical representation of these tests). However, as this configuration of psychological processes has not been tested before, direct relationships will also be tested.

## 2. Materials and Methods

### 2.1. Recruitment

Dog and cat owners were recruited separately. Dutch dog owners were invited to participate in research concerning the human–dog relationship, by a link shared on social media (mainly Facebook), between January and April 2020. Cat owners were recruited between June and July 2020 by means of a link shared on social media (Facebook and Twitter) and several cat-related Dutch newsletters. Participants of the “Purrdoctor Cat Cohort” received an e-mail with the link to the survey.

### 2.2. Procedure

This study has been approved by the University of Groningen’s Ethics Committee of Psychology (PSY-1920-S-0061 dogs, PSY-1920-S-0497 cats). The participants filled out an online questionnaire in Dutch, that was generated and hosted at a data collection platform (Qualtrics). Before filling out the questionnaire participants were presented with the following information: a short description of the questionnaire including approximate duration; data processing; privacy rights; statements that participation was voluntary and that cessation of participation was without penalty; and the researcher´s contact information.

### 2.3. Measurements

#### 2.3.1. Background Variables

Participants’ demographic information (sex, age, and education) was collected and dichotomized (sex: female vs male; education: low vs medium education/ high education; according to the Dutch educational system). Age was left as a continuous variable. Because knowledge about pets can affect the degree of anthropomorphization, e.g., [21], we examined the level of species-specific knowledge by asking if participants had a profession related to cats or dogs (yes vs. no). Participants who had multiple pets in their home were asked to answer the questions with only one cat/dog in mind. We asked the name of the pet which showed up automatically in the survey, integrated in the questions. Background information on breed, sex, and age of the pet, and where and how long ago the pet had been acquired, and the reasons for acquiring the pet, was also gathered but because this information is not pertinent to the present research questions, it will not be presented further.

#### 2.3.2. Measures of Anthropomorphic Tendencies

As a general measure of anthropomorphic attributions to animals we used the *Individual Differences in Anthropomorphism Questionnaire* (IDAQ) [40] which asks participants to which extent five animals (insect, fish, reptile, cow, and cheetah) have free will, emotions, intentions, consciousness and a mind of their own, on an eleven-point scale from not at all (1) to very much (11), of which the average item score was used as the scale score. This scale has been validated [40] and often used by other authors [41,42,43,44]. The reliability of this scale (measured using Cronbach’s alpha) in the present study was good (α = 0.79).

However, as we were interested in the human–pet relationship, and asked people about perceptions of, and interactions with, a pet they currently lived with, we believed that the IDAQ was a too general measure of anthropomorphic tendencies. Moreover, in self-report questionnaires the question format is crucial to the validity of the measurement. When asking pet owners whether they think their pet is able to understand human language, they may be affirmative because they think the animal understands it “in its own way”. For this reason, we used an anthropomorphization comparison answering format [45] in which we asked owners to reflect on the degree their pet could execute several cognitive and social abilities “similar to those of humans”. It is of interest to see to how well our comparative measure of anthropomorphization predicts the outcomes compared to the commonly used IDAQ.

The attribution of abilities as measured by our new measure comparative anthropomorphisation (COANT) was assessed by measuring the attribution of higher order cognitive abilities such as reasoning, counting, making decisions, thinking about the future and the past, social abilities such as deceiving on purpose, taking revenge, understanding human emotions, understanding human language and emotions such as fear, happiness, compassion, jealousy, and self-pity. These abilities were rated on an eight-point scale: no (0); yes, but certainly not similar to humans (1); yes, slightly similar to humans (2); yes, moderately similar to humans (3); yes, similar to humans (4); yes, very similar to humans (5); yes, almost the same as humans (6); and yes, exactly like humans (7), and the average item score was used as the scale score (α = 0.95).

The perception of the pet in a human social role, the *Pet Role Perception* (PRP), was assessed by a scale consisting of four items inspired by Albert and Bulcroft [26] and Vink and Dijkstra [27]: “my dog is like a child to me”, “love for animals is real love”, “my dog is a full member of the family” and “my dog is a true friend”, which were scored on a seven-point scale: strongly disagree (1), disagree (2), slightly disagree (3), neither agree nor disagree (4), slightly agree (5), agree (6), and strongly agree (7). The average item score was used as the scale score (α = 0.77).

#### 2.3.3. Relationship Outcomes

*Communication behavior* was assessed using a scale with five items inspired by Fournier et al. [46] and Frank [47] that measured forms of physical and communicative contact associated with affection. The owner is asked to report how often he/she (a) pets the dog/cat, (b) hugs the dog/cat, (c) kisses the dog/cat, and (d) talks to the dog. Items were rated on a 7-point scale: never (1), rarely (2), sometimes (3), regularly (4), often (5), very often (6), and always (7), and the average item score was used as the scale score (α = 0.79).

*Making-up behavior* was assessed with six items [29]. The items asked how often people tried to mend the relationship with the pet (for example, when they feel a little bit guilty for situations like not giving the pet enough attention): “giving extra food”, “giving treats”, “giving a new toy”, “giving extra attention”, “giving extra cuddles”, and “apologizing”. The items were rated on a rated on a 7-point scale: never (1), rarely (2), sometimes (3), regularly (4), often (5), very often (6), and always (7), and the average item score was used as the scale score (α = 0.799).

*Social support* was assessed by a scale with ten items, inspired by two human social support scales [48,49]. Example items are: “I can count on my pet when things go wrong” or “my pet gives me the emotional help and support that I need”, rated on a seven-point scale: strongly disagree (1), disagree (2), slightly disagree (3), neither agree nor disagree (4), slightly agree (5), agree (6), and strongly agree (7) and the average item score was used as the scale score (α = 0.95).

### 2.4. Statistical Analyses

Firstly, descriptive and correlational data are presented. Secondly, the direct effects are tested. Thirdly, the mediation effects are tested. Fourthly, pet type is introduced, and moderated mediation is tested. Age, gender, educational level, and working professionally with animals were studied as potential covariates. The statistical software SPSS Statistics 27 for Windows was used (Armonk, NY, USA) for descriptive data, and main and moderation effects. Mediation and moderated mediation were performed using model 4 and 7 of the SPSS 25 PROCESS module of Hayes, respectively, with 5000 bootstraps, and all the variables were transformed into z-scores.

## 3. Results

### 3.1. Participant Selection

Of the 702 people who entered the electronic questionnaire, 640 (dogs *n* = 378, cats *n* = 262) provided complete data on the six core psychological and behavioral variables. To decide about the final study sample, it was studied whether covariates had to be included in the main analyses. Four potential covariates in the analyses of variance were studied: gender, level of education, age, and working professionally with pets or not. Correlations of these variables with the six psychological and behavioral values were computed (Pearson for age and Spearman for the others) in the 640-participant sample. Age and level of education were especially significantly related to all six and four psychological and behavioral variables, respectively, with the largest correlation being 0.25. It was decided to include both variables as covariates on all below analyses of variance. Although the study sample size now further decreased to 527 participants with complete data (332 dog owners and 195 cat owners), it was estimated that it still would provide sufficient statistical power.

### 3.2. Participant Characteristics

With regard to level of education 43% of the participants (*n* = 527) had a lower or medium level of education and 57% a high level. The mean age was 47 (SD = 13), and 88% was female and 12% male (1 “rather not say”). Twenty-six percent of the participants worked professionally with pets (shelter volunteer, groomer, veterinarian, behaviorist, etc.; five missing). The pet’s sex was 50% female, 39% was between 1 and 4 years of age, 29% between 5 and 8, and 22% between 9 and 14, and the duration of the presence of most pets fell in the categories 2 to 5 years (30%) and 5 to 10 years (31%).

### 3.3. Differences between Cats and Dogs

First, we examined whether cats and dogs owners differed on demographic variables, with Chi-square tests for categorical variables and an F-test for owner age. This showed that there are significant differences (*p* < 0.05) between owners (statistics not shown): cats were more often owned by females, cat owners more often had a high level of education, dog owners more often had a pedigree, on average cats were older than dogs, and (hence) cats were more often owned for more than 10 years. Secondly, cat and dog owners were compared on the predictors and outcome measures (Table 1). Analyses of variance showed that cats and dog owners differed significantly on all measures (*p* < 0.05), except for making up. On the remaining five variables, cat owners scored higher on the IDAQ, but dog owners scored higher on the other four variables.

### 3.4. Correlations

Pearson correlations (Table 1) were computed to show the univariate relationships between the six variables. The results show that comparative anthropomorphization was significantly related to all three outcome measures (0.25–0.38), while the IDAQ was only weakly related to one of the three (0.13). Furthermore, comparative anthropomorphization and IDAQ were both significantly related to pet role perception, but with correlations of different magnitude (0.38 and 0.16, respectively). Both measures of anthropomorphization correlated significantly, but the 0.27 correlation suggests they differ substantially. The correlations among the three outcome measures ranged from 0.43 to 0.88, possibly revealing a common ground.

### 3.5. Direct Effects

To test direct effects, the three psychological variables were entered in three multiple regression analyses, one for each of the three outcome variables (including age and level of education as covariates). In the model predicting making-up, comparative anthropomorphization and pet role perception both were significant, B = 0.10, *p* < 0.02, 95% CI 0.02 to 0.19, and B = 0.36, *p* < 0.001, 95% CI 0.28 to 0.45, respectively. The model explained 22.4% of the variance in making up. In the model with social support as dependent variable, again comparative anthropomorphization and pet role perception both were significant, B = 0.13, *p* = 0.002, 95% CI 0.05 to 0.22, and B = 0.67, *p* < 0.001, 95% CI 0.59 to 0.75, respectively. The model explained 43.9% of the variance in social support. In the model with communication behavior as dependent variable, only pet role perception was significant, B = 0.70, *p* < 0.001, 95% CI 0.60–0.79. The model explained 43.9% of the variance in communication behavior. Thus, pet role perception predicted all three outcome variables, comparative anthropomorphization did not predict communication behavior, while the IDAQ was not related to any outcome variable.

### 3.6. Mediation Effects

**Figure 2:**
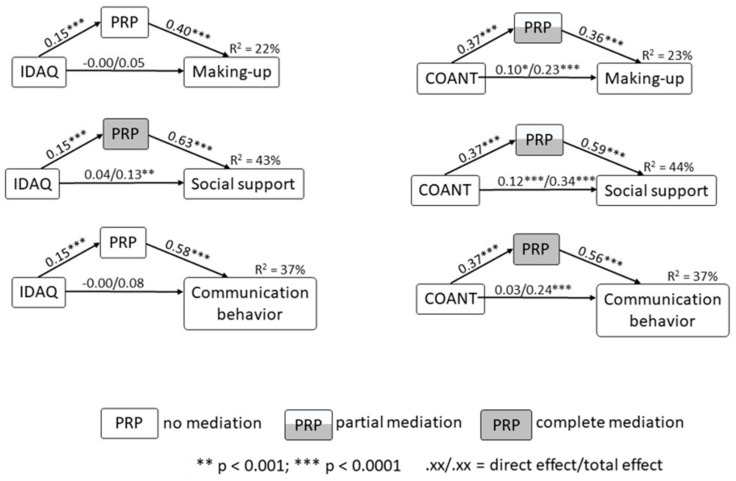
Direct and mediation effects of IDAQ (Individual Differences in Anthropomorphism Questionnaire) and comparative anthropomorphization (COANT) on Social Support and Communication behavior.

The PROCESS model 4 of Hayes was used to test the data on mediation (including age and level of education as covariates). For each of the three dependent variables (Y), two models were tested; one with the IDAQ as predictor (X1), and one with comparative anthropomorphization as predictor (X2), while in both models pet role perception was examined as potential mediator (M). The diagrams depicted in Figure 2 illustrate the findings. Regarding the relationship of IDAQ with the three outcome variables, only with regard to social support the mediation was complete, significant and meaningful, (coeff. = 0.09, CI 0.04 to 0.15). With regard to making up and communication behavior the bootstrap test was significant but the IDAQ had no relationship with both that could be mediated. Regarding the relationship of the COANT with the three outcome variables, the bootstrap test showed significant mediation with regard to all three outcome variables. With regard to making up, the mediation was significant and partial, (coeff. = 0.13, CI 0.09 to 0.16). With regard to social support, the mediation was significant and partial, (coeff. = 0.22, CI 0.17 to 0.28). With regard to communication behavior, the mediation was significant and complete, (coeff. = 0.21, CI 0.16 to 0.26). See Figure 2 for a graphical representation of the results.

### 3.7. Moderated Mediation

The PROCESS model 7 of Hayes was used to test the data on moderation mediation (including age and level of education as covariates). That is, if the above significant mediations might differ for cat and dog owners. Again, for each of the three dependent variables (Y), the IDAQ and comparative anthropomorphization were used as the predictor (X), PRP as mediator (M), while the relationship between X and M might be moderated by type of pet (cat or dog; Z). With regard to mediation of the IDAQ by pet role perception, all three moderated mediation tests were not significant. With regard to mediation of the COANT by pet role perception, all three moderated mediation tests were significant, and all mediations, in cats and in dogs, were significant, but in cats the relationship was stronger compared to dogs: making up: moderated mediation coeff. = 0.10, CI 0.04 to 0.17, the coefficient in cats was 0.21, in dogs 0.11; social support: moderated mediation coeff. = 0.17, CI 0.06 to 0.28, the coefficient in cats was 0.34, in dogs 0.17; and communication behavior: moderated mediation coeff. = 0.16, CI 0.05 to 0.27, the coefficient in cats was 0.32, in dogs 0.16.

## 4. Discussion

The goal of this study was to examine to what extent anthropomorphization is associated with owners’ relationship behaviors (communication and making up) and experience of social support, and whether these associations were mediated by the perception of the social role of the pet. Possible differences between cat and dog owners were examined. Our results reveal several relevant patterns.

The results from the correlational analyses are in line with our hypotheses, showing significant correlations in the expected directions. The outcome measures had significant relationships with each other (from 0.43 to 0.88), which is in line with having a common ground (i.e., certain perceptions of the pet). The correlations also showed that the two measures of anthropomorphization, the traditional IDAQ and our comparative measure COANT, were related significantly to each other (0.27), thereby partly validating our new comparison scales with the IDAQ. Still, the low magnitude of the correlation suggests that they also assess different constructs (see below).

The multiple linear regression analyses testing direct effects, controlled for the overlap between the two anthropomorphization measures, and it showed that the IDAQ had no unique relationship anymore with the outcome measures while the COANT was related to making up and social support, but not to communication behavior. The more pet owners rated the experience/presence of mental abilities of their pet to be similar to that of humans, the more they displayed making-up behavior towards their pet, and the stronger they experienced social support from their pet. Although no causal relationships can be inferred from our data, making-up behavior is motivated and shaped by people’s (anthropomorphic) perceptions: Why bother “repairing” a relationship when the other would not be equipped to understand at least the making-up behavior in part. Similarly, social support can be conceptualized as being (partly) determined by anthropomorphization as ‘’People create agents of social support by anthropomorphizing their pets” [22], p. 148. Our results indicate that in order to engage in communication behavior (e.g., petting, kissing, talking) it is not necessary to attribute human abilities to the pet. It may be that the perception that the pet at the least “experiences something” in reaction to these behaviors is sufficient to generate the behavior in the pet owner when they have a social need.

A central test was whether the relationship of the two measures of anthropomorphization with the three outcomes was mediated by the perception of the pet’s social role; the extent to which the pet was perceived in terms of being a child, a friend, a family member, and provider of love [17,26,27]. The correlational analyses showed that both measures of anthropomorphization were indeed significantly, albeit weakly, related to the perception of the social role of the pet (0.16 IDAQ and 0.38 COANT) and the correlations of the perception of the social role of the pet with the three outcomes measures were all significant and of substantial magnitude (0.43 to 0.65). Of the six tests of mediation, four were significant, which supports our theoretically conceptualized sequence of processes: anthropomorphization leads to perception of the pet’s social role, which leads to engaging in communication and making-up behaviors and the experience of social support. In two of these analyses, the mediation was partly: our measure of anthropomorphization was still related to the outcomes directly, independent of social role perception.

Some other relevant observations can be made from the mediation models. First, the IDAQ had the weakest (often non-significant) relationship with the three outcomes which may be due to the IDAQ’s general formulations of the questions, while our comparative anthropomorphization measure of cognitive/social abilities was related to all three outcomes. Second, the variances in the three behaviors explained by the measures of anthropomorphization and pet role perception ranged from 22% to around 44%. Although much variance is not accounted for, these percentages are comparable to the 20% prediction by psychological variables of human health behaviors that has been found in a meta-analysis [50]. These percentages reveal that other causes must be considered to further understand people’s behaviors towards their pets. In this light it is important to realize that anthropomorphization and social role perceptions in themselves do not motivate relationship behaviors. That is, much variance in the measures of behavior is probably motivated by the social needs of the owner. The explained variance in social support was above 40%, suggesting larger effects of the predictors, and a more complete model underlying the origin of social support experience. Another observation is that pet role perception has the strongest relations with the outcomes. Thus, in a causal interpretation, the relationship outcomes were more strongly determined by the perceptions of the social role of the pet than by attribution of mental abilities.

A strength of the present study is that cat and dog owners were included in samples of substantial size. Our hypothesis that cats and dogs behave in different ways, which are reflected in the owners’ perceptions of their pets, was partly confirmed as we found some significant differences. Dog owners attributed more mental abilities to their pet compared to cat owners, they perceived their dog in a more social role, communicated more with their dog and reported more social support from their dog. These differences may reflect the species-specific evolution and domestication of cats and dogs that can nowadays still be observed by owners. As cats are facultatively social, and descendants of solitary hunters [51], social cognitive skills might be less evolved in cats than in group-living animals such as humans and dogs, for whom social skills are necessary for survival. Moreover, dogs were among the first domesticated animals [52], partly due to their social nature, but also because of their ancestors’ willingness (or ability) to change behavior in response to human behavior [53]. Cats had a much shorter and less function-driven domestication history with humans [54] than dogs, and humans had less control in shaping ‘social communication skills’ in cats.

Our results show that dog owners anthropomorphize their dog more than the cat owners in our sample (indicated by a higher attribution of mental abilities and seeing the pet more often in a human-like social role). This is consistent with Vonk who showed that the strength of the owner-pet bond was associated with a higher tendency to mental abilities to pets and that this tendency is higher for dogs than for cats [55]. which might be due to a higher symmetry in social behaviors of humans and dogs compared to humans and cats. Thus, the actual differences in species and their history with humans was partly reflected in how owners perceive their pets. Not only these perceptual states differed, also the psychological processes that are assumed to underlie the relationship outcome differed for cat and dog owners: In cat owners, the effect of anthropomorphization was more strongly mediated by the perceived pet role perception compared to dog owners. This may suggest that in cat owners, pet role perception is more central in producing relationship outcomes than in dog owners.

The measures of anthropomorphization are a central aspect in this study. The IDAQ was chosen because it is the most widely used measure, and has been applied in many contexts [40,56,57]. Although only the subscale with regard to the attribution of mental abilities in different animal species was used, this measure was not optimal for assessing psychological processes that operate in close relationships with a specified individual cat or dog. In the relationship with one’s own pet, it may not be relevant whether mental activities are attributed to other types of animals, as different animal species are anthropomorphized to different degrees [45,58]. The format of our new anthropomorphization measure referred to the owner’s pet with the name of the pet (indicated by the owner at the start of the survey) inserted into the question. In addition to this personalized format, the new measure applied a comparative format. Anthropomorphization is about attributing human (mental) abilities, but we noticed from interviews about emotions of animals that pet owners sometimes acknowledged that animals had certain emotions (e.g., feeling of guilt), but “in their own way” or “only resembling those of humans”. Therefore, in line with Eddy et al. [45], the present item format was about the extent to which the pet had the specified ability “similar to that of humans”. Answering “no” could mean that the pet was believed to have the specified ability, but not in a similar way as humans. The directions of the relationships of our new scale, with the IDAQ but also with the other measures, were all in expected directions, thereby supporting their validity.

Several limitations of the present study need to be considered. Regarding the participant sample, people with a low educational level were under-represented (7%) and people with a high educational level were overrepresented (58%). Furthermore, as is common in animal-related research, women were over represented, despite actively encouraging men to participate. As women in general, seem to have a more positive attitude towards animals [59], seem to attach more to animals [60], and have more empathy for living beings [61], our results might be less applicable to male pet owners. Furthermore, also with regard to the compositions of the cat owners versus dog owner samples, a relevant selection may have occurred. For example, the percentage of women was higher in our cat sample, while women have a higher tendency to attribute human social characteristics (e.g., empathy) to cats (compared to men) [24]. Although these limitations may undermine the generalizability, the tests for potential covariates showed that the relationships with level of education and gender were only small. In addition, as is common in studies about human–animal relationships, this research unintentionally especially attracted highly engaged pet owners, which may have biased our results. Still, there was substantial variance in most measures, suggesting that the effects of self-selection on the core variables were limited. The high standard deviations in our anthropomorphic measures imply that the variability in owners’ beliefs about their pets’ mental states vary to a large extent. This finding is consistent with previous research about complex cognition and emotional experiences of pets [62,63]. However, although the psychological processes related to anthropomorphic thinking can differ significantly between individuals, Waytz et al. [40], showed that individuals’ tendency to attribute human-like abilities to non-human entities changes little across time. One last characteristic of the present study to be mentioned is related to the scales that were used. Although most scales were developed for this study, the items have all been used before in earlier research on anthropomorphization [46,64,65,66]. The present study aimed to carefully distinguish between different constructs, for example, potential psychological causes of behavior (measures of anthropomorphsation and of pet social role perception) versus behavioral manifestations in the relationship and experience of social support.

## 5. Conclusions

Understanding the human–animal relationship is relevant because it is of great importance to people and their wellbeing but should, from an ethical point of view, not burden animals. Although some degree of anthropomorphism might be necessary to experience a connection with an animal [21], owners need to keep in mind that cats and dogs are not small humans; they are different species with their own species-specific needs. Projecting one’s own human mental abilities to other humans may support adequate communication and a pleasant relationship development; however, the effects of wrongly projecting human abilities to animals may negatively influence a sustainable, and for both sides beneficial, relationship between humans and animals. Understanding how anthropomorphization influences human perceptions of animals is essential in understanding the human–animal relationship. Although the old-school use of the term anthropomorphization has been criticized as a dead end [66], in the contemporary scientific approach it has been conceptualized as a psychological process, that serves and influences social interactions [36]. This is a promising perspective on anthropomorphization and related perceptions that may shed new light on how people relate to the broad class of nonhuman living creatures that they are surrounded by.

## Figures and Tables

**Table 1:** Averages scores and correlations of predictors and outcome measures.

	*n* = 527	Cats (M, SD)	Dogs (M, SD)	1	2	3	4	5
1	IDAQ	5.01 (2.11)	4.74 (2.25)					
2	Comparative anthropomorphization	1.34 (0.55)	1.72 (1.05)	0.27 **				
3	Pet Role Perception	5.27 (1.35)	5.74 (1.09)	0.16 **	0.38 **			
4	Making up	3.30 (0.89)	3.28 (1.07)	0.08	0.27 **	0.43 **		
5	Social Support	4.83 (1.17)	5.37 (1.05)	0.13 **	0.33 **	0.65 **	0.43 **	
6	Communication behavior	5.12 (1.24)	5.49 (1.21)	0.08	0.25 **	0.60 **	0.43 **	0.88 **

Note: ** *p* ≤ 0.001.

## Data Availability

The data presented in this study is not openly available.

## References

[1] Fox R. (2006). Animal behaviors, post-human lives: Everyday negotiations of the animal-human divide in pet-keeping. Soc. Cult. Geogr..

[2] Charles N., Davies C.A. (2008). My family and other animals: Pets as kin. Sociol. Res. Online.

[3] Bekoff M. (2007). Animals Matter: A Biologist Explains Why We Should Treat Animals with Compassion and Respect.

[4] Mason J., Tipper B. (2008). Being Related: How children define and create kinship. Childhood.

[5] Bowlby J. (1973). Attachment and loss. Attachment and Loss: Volume II: Separation, Anxiety and Anger.

[6] Dunbar R.I.M. (2012). Bridging the bonding gap: The transition from primates to humans. Phil. Trans. R. Soc. B.

[7] Endenburg N., Bouw J. (1994). Motives for acquiring companion animals. J. Econ. Psychol..

[8] Cromer L., Barlow M.R. (2013). Factors and Convergent Validity of The Pet Attachment and Life Impact Scale (PALS). Hum.-Anim. Interact. Bull..

[9] Serpell J.A. (1996). Evidence for an association between pet behavior and owner attachment levels. Appl. Anim. Behav. Sci..

[10] Johnson E., Volsche S. (2021). COVID-19: Companion Animals Help People Cope during Government-Imposed Social Isolation. Soc. Anim..

[11] Zasloff R.L. (1996). Measuring attachment to companion animals: A dog is not a cat is not a bird. Appl. Anim. Behav. Sci..

[12] Sable P. (1995). Pets, attachment, and well-being across the life cycle. Soc. Work.

[13] Cohen S.P. (2002). Can pets function as family members? West. J. Nurs. Res..

[14] Walsh F. (2009). Human-animal bonds I: The relational significance of companion animals. Fam. Process.

[15] Stammbach K.B., Turner D.C. (1999). Understanding the Human—Cat Relationship: Human Social Support or Attachment. Anthrozoös.

[16] Hill R.P., Gaines J., Wilson R.M. (2008). Consumer behavior, extended-self, and sacred consumption: An alternative perspective from our animal companions. J. Bussiness Res..

[17] Bouma E.M.C., Reijgwart M.L., Dijkstra A. (2022). Family member, best friend, child or ‘just’a pet, owners’ relationship perceptions and consequences for their cats. Int. J. Environ. Res. Public Health.

[18] Rynearson E.K. (1978). Humans and pets and attachment. Br. J. Psychiatry.

[19] Van Houtte B.A., Jarvis P.A. (1995). The role of pets in preadolescent psychosocial development. J. Appl. Dev. Psychol..

[20] Holbrook M.B., Stephens D.L., Day E., Holbrook S.M., Strazar G. (2001). A collective stereographic photo essay on key aspects of animal companionship: The truth about dogs and cats. Acad. Mark. Sci. Rev..

[21] Epley N., Waytz A., Cacioppo J.T. (2007). On seeing human: A three-factor theory of anthropomorphism. Psychol. Rev..

[22] Epley N., Waytz A., Akalis S., Cacioppo J.T. (2008). When we need a human: Motivational determinants of anthropomorphism. Soc. Cogn..

[23] Konok V., Kosztolányi A., Rainer W., Mutschler B., Halsband U., Miklósi Á. (2015). Influence of Owners’ Attachment Style and Personality on Their Dogs’ (*Canis familiaris*) Separation-Related Disorder. PLoS ONE.

[24] Pongrácz P., Szapu J.S. (2018). The socio-cognitive relationship between cats and humans—Companion cats (*Felis catus*) as their owners see them. Appl. Anim. Behav. Sci..

[25] Szánthó F., Miklósi Á., Kubinyi E. (2017). Is your dog empathic? Developing a Dog Emotional Reactivity Survey. PLoS ONE.

[26] Albert A., Bulcroft K. (1988). Pets, families, and the life course. J. Marriage Fam..

[27] Vink L., Dijkstra A. (2019). The Psychological processes involved in the development of a high-quality relation with one’s dog. Hum.-Anim. Interact. Bull..

[28] Barsalou L.W. (2014). Cognitive Psychology: An Overview for Cognitive Scientists.

[29] Cryder C.E., Springer S., Morewedge C.K. (2012). Guilty feelings, targeted actions. Personal. Soc. Psychol. Bull..

[30] Van Splunter M.A. (2007). Nationale Hondenwijzer.

[31] Uchino B.N. (2009). Understanding the Links between Social Support and Physical Health: A Life-Span Perspective with Emphasis on the Separability of Perceived and Received Support. Perspect. Psychol. Sci..

[32] Reniers P.W.A., Declercq I.J.N., Hediger K., Enders-Slegers M.-J., Gerritsen D.L., Leontjevas R. (2022). The role of pets in the support systems of communitydwelling older adults: A qualitative systematic review. Aging Ment. Health.

[33] Hill L., Winefield H., Bennett P. (2020). Are stronger bonds better? Examining the relationship between the human–animal bond and human social support, and its impact on resilience. Aust. Psychol..

[34] Turner D.C., Rieger G., Gygax L. (2003). Spouses and cats and their effects on human mood. Anthrozoos.

[35] Kurdek L.A. (2009). Pet dogs as attachment figures for adult owners. J. Fam. Psychol..

[36] Greene J.O. (1984). A cognitive approach to human communication: An action assembly theory. Commun. Monogr..

[37] Greene J.O. (2013). A second generation action assembly theory. Message Production.

[38] Mattiello E., Ritt-Benmimoun V., Dressler W.U. (2021). Asymmetric use of diminutives and hypocoristics to pet animals in Italian, German, English, and Arabic. Lang. Commun..

[39] Most Popular Pets by Country. Website World Population. https://worldpopulationreview.com/country-rankings/most-popular-pets-by-countr.com.

[40] Waytz A., Cacioppo J., Epley N. (2010). Who sees human? The stability and importance of individual differences in anthropomorphism. Perspect. Psychol. Sci..

[41] Keefer L.A. (2016). Is anybody out there? Trait anthropomorphism predicts the psychological benefits of a favorite belonging. J. Individ. Differ..

[42] Letheren K., Kuhn K.A.L., Lings I., Pope N.K.L. (2016). Individual difference factors related to anthropomorphic tendency. Eur. J. Mark..

[43] Severson R.L., Woodard S.R. (2018). Imagining others’ minds: The positive relation between children’s role play and anthropomorphism. Front. Psychol..

[44] Tam K.P. (2019). Anthropomorphism of nature, environmental guilt, and pro-environmental behavior. Sustainability.

[45] Eddy T.J., Gallup G.G., Povinelli D.J. (1993). Attribution of cognitive states to animals: Anthropomorphism in comparative perspective. J. Soc. Issues.

[46] Fournier A.K., Berry T.D., Letson E., Chanen R. (2016). The human–animal interaction scale: Development and evaluation. Anthrozoös.

[47] Frank L.K. (1957). Tactile communication. Genet. Psychol. Monogr..

[48] Van Sonderen E. (1993). Het Meten van Aspecten van Sociale Steun Met de Sociale Steun Lijst Interacties (SSL-I) en Social Steun Lijst Discrepanties (SSL-D): Een handleiding. Noordelijk Centrum Voor Gezondheidsvraagstukken.

[49] Zimet G.D., Dahlem N.W., Zimet S.G., Farley G.K. (1988). The Multidimensional Scale of Perceived Social Support. J. Personal. Assess..

[50] McEachan R.R.C., Conner M., Taylor N.J., Lawton R.J. (2011). Prospective prediction of health-related behaviours with the theory of planned behaviour: A meta-analysis. Health Psychol. Rev..

[51] Bradshaw J.W.S. (2016). Sociality in cats: A comparative review. J. Vet. Behav..

[52] Clutton-Brock J. (1999). A Natural History of Domesticated Mammals.

[53] Horowitz A. (2014). Domestic Dog Cognition and Behavior.

[54] Driscoll C.A., Menotti-Raymond M., Roca A.L., Hupe K., Johnson W.E., Geffen E., Harley E.H., Delibes M., Pontier D., Kitchener A.C. (2007). The Near East origin of cat domestication. Science.

[55] Vonk J. (2023). The Bonds between Us: Stronger Bonds Predict Greater Attribution of Companion Animal Socio-cognitive Skills. Appl. Anim. Behav. Sci..

[56] Cullen H., Kanai R., Bahrami B., Rees G. (2014). Individual differences in anthropomorphic attributions and human brain structure. Soc. Cogn. Affect. Neurosci..

[57] Neave N., Jackson R., Saxton T., Hönekopp J. (2015). The influence of anthropomorphic tendencies on human hoarding behaviours. Personal. Individ. Differ..

[58] Higgs M.J., Bipin S., Cassaday H.J. (2020). Man’s best friends: Attitudes towards the use of different kinds of animal depend on belief in different species’ mental capacities and purpose of use. R. Soc. Open Sci..

[59] Herzog H.A., Betchart N.S., Pittman R.B. (1991). Gender, sex role orientation, and attitude towards animals. Anthrozoös.

[60] Quinn A.C. (2005). An Examination of the Relations between Human Attachment, Pet Attachment, Depression, and Anxiety. Ph.D. Dissertation.

[61] Baron-Cohen S., Wheelwright S. (2004). The empathy quotient: An investigation of adults with Asperger syndrome or high functioning autism, and normal sex differences. J. Autism. Dev. Disord..

[62] Arahori M., Kuroshima H., Hori Y., Takagi S., Chijiiwa H., Fujita K. (2017). Owners’ view of their pets’ emotions, intellect, and mutual relationship: Cats and dogs compared. Behav. Process..

[63] Paul H., Morris P.H., Doe C., Godsell E. (2008). Secondary emotions in non-primate species? Behavioural reports and subjective claims by animal owners. Cogn. Emot..

[64] Howell T.J., Toukhsati S., Conduit R., Bennett P. (2013). The perceptions of dog intelligence and cognitive skills (PoDIaCS) survey. J. Vet. Behav..

[65] Kupsala S., Vinnari M., Jokinen P., Räsänen P. (2016). Public perceptions of mental capacities of nonhuman animals: Finnish population survey. Soc. Anim..

[66] Wynne C.D. (2007). What are animals? Why anthropomorphism is still not a scientific approach to behavior. Comp. Cogn. Behav. Rev..

